# Supplementary material for: Quantitative Approach to Quality Review of Prenatal Ultrasound Examinations: Estimated Fetal Weight and Fetal Sex
Source: J Clin Med. 2024 Nov 16;13(22):6895. doi: 10.3390/jcm13226895 (PMC11594779; doi:10.3390/jcm13226895)
Supplement: Supplementary file 1 [file jcm-13-06895-s001.zip › S1 - Derivation and validation of Formula.pdf]

Supplementary File 1
Derivation and Validation of Formula for Birth Weight Prediction

S1.1 Derivation of Formula

S1.1.1 Abbreviations and symbols used

BW = birth weight (grams)
$BW_{predicted}$ = predicted birth weight (grams)
EFW = estimated fetal weight (grams) at time of ultrasound
exp[x] = e^x where e is the Euler constant, approximately 2.78218
GA_{birth} = gestational age at birth (exact weeks, i.e., weeks + decimal fraction)
GA_{us} = gestational age at time of ultrasound (exact weeks)
Ln[x] = natural logarithm of x
MFW_{birth} = mean standard fetal weight GA_{birth} (grams) (from Hadlock et al, [36])
MFW_{us} = mean standard fetal weight at GA_{us} (grams) (from Hadlock et al, [36])
$Pctile_{predicted}$ = predicted fetal weight percentile at birth
$Pctile_{efw}$ = estimated fetal weight percentile at time of ultrasound
SD_{birth} = standard deviation of MFW_{birth} (grams) (from Hadlock et al, [36])
SD_{us} = standard deviation of MFW_{us} (grams) (from Hadlock et al, [36])
$Z_{predicted}$ = z-score of $BW_{predicted}$
Z_{efw} = z-score of EFW
Ln(x) = natural (base-e) logarithm of x
• = multiplication symbol
|x| = absolute value of x

S1.1.2 Derivation of formula

The method of Mongelli & Gardosi [24] assumes that the ratio of EFW to MFW_{us} remains constant over time and should therefore equal the ratio of BW to MFW_{birth}. This can be expressed as:

$$EFW/MFW_{us} = BW_{predicted}/MFW_{birth}$$

And solving for $BW_{predicted}$:

$$BW_{predicted} = EFW \bullet (MFW_{birth}/MFW_{us})$$

For MFW values, Mongelli and Gardosi [24] cite Hadlock et al. [36]. The text of Mongelli and Gardosi uses median rather than mean, but the Hadlock formula gives means. The distinction is irrelevant because MFW is normally distributed, so the mean and median are identical. Hadlock's MFW is a function GA, f(GA), of the form:

$$Ln(MFW) = f(GA)$$
where f(GA) is $(a \bullet GA^2 + b \bullet GA + c)$
and a = –0.00354, b = 0.332, and c = 0.578

Taking the antilog of both sides:

$$MFW = \exp[f(GA)]$$

Substituting this into the formula for $BW_{predicted}$ above:

$$BW_{predicted} = EFW \bullet ([\exp(f(GA_{birth})] \;/\; \exp[f(GA_{us})])$$

Taking the natural log of both sides:

$$Ln(BW_{predicted}) = Ln(EFW) + f(GA_{birth}) - f(GA_{us})$$

Substituting the values for f(GA):

$$Ln(BW_{predicted}) = Ln(EFW) + (a\bullet GA_{birth}^2 + b\bullet GA_{birth} + c) - (a\bullet GA_{us}^2 + b\bullet GA_{us} + c)$$

Redistributing and simplifying:

$$Ln(BW_{predicted}) = Ln(EFW) + a\bullet(GA_{birth}^2 - GA_{us}^2) + b\bullet(GA_{birth} - GA_{us})$$

And taking the anti-log of both sides:

$$BW_{predicted} = \exp[Ln(EFW) + a\bullet(GA_{birth}^2 - GA_{us}^2) + b\bullet(GA_{birth} - GA_{us})]$$

$$= EFW \bullet \exp[a\bullet(GA_{birth}^2 - GA_{us}^2) + b\bullet(GA_{birth} - GA_{us})]$$

$$= EFW \bullet \exp[- 0.00354 \bullet (GA_{birth}^2 - GA_{us}^2) + 0.332 \bullet (GA_{birth} - GA_{us})]$$

Or

$$BW_{predicted} = EFW \bullet e^x$$

$$\text{where } x = - 0.00354 \bullet (GA_{birth}^2 - GA_{us}^2) + 0.332 \bullet (GA_{birth} - GA_{us})$$

This is the formula in the article.

The formula does not depend on which formula is used for EFW. We used Hadlock (1985), third formula in Table II, but any other EFW formula can be used instead, depending on local practice.

S1.1.3 Equivalence of assuming of constant EFW percentile or constant z-score

The method of Mongelli and Gardosi [24] can be shown to be identical to assuming that EFW percentile remains constant from ultrasound to birth. In other words:

$$Pctile_{predicted} = Pctile_{efw}$$

Percentiles have a 1:1 correspondence with z-scores, so this is equivalent to stating that

$$Z_{predicted} = Z_{efw}$$

Substituting the formula for z-score:

$$(BW_{predicted} - MFW_{birth})/SD_{birth} = (EFW - MFW_{us})/SD_{us}$$

According to Hadlock et al [36], SD is 0.12 (12% of MFW), so substituting that into formula above:

$$(BW_{predicted} - MFW_{birth})/(0.12 \bullet MFW_{birth}) = (EFW - MFW_{us})/(0.12 \bullet MFW_{us})$$

Multiplying both sides of equation by 0.12:

$$(BW_{predicted} - MFW_{birth})/MFW_{birth} = (EFW - MFW_{us})/MFW_{us}$$

Distributing the numerators and simplifying:

$$BW_{predicted}/MFW_{birth} - MFW_{birth}/MFW_{birth} = EFW/MFW_{us} - MFW_{us}/MFW_{us}$$

$$BW_{predicted}/MFW_{birth} - 1 = EFW/MFW_{us} - 1$$

$$BW_{predicted}/MFW_{birth} = EFW/MFW_{us}$$

And solving for $BW_{predicted}$

$$BW_{predicted} = EFW \bullet (MFW_{birth}/MFW_{us})$$

This is identical to the second equation in Section S1.1.2. So Mongelli and Gardosi's assumption of a constant weight ratio over time is identical to assuming a constant percentile or a constant z-score.

S1.2 Validation of Formula

The accuracy of BW predictions made using the formula is summarized in Table S1. For latencies <12 wks, 90% of predictions or more were within ±20% of birthweight and the rate of errors ≥30% was 3% or less. Accuracy was higher in analyses restricted to the last exam before birth (lower half of Table). Accuracy decreased with increasing latency.

We restricted the quality review to exams with latency <12 wks because exams with latency ≥12 weeks had lower accuracy (more than 10% of exams with absolute error ≥20%).

Table S1. Accuracy of birth weight predictions at different latencies

Latency	N	Percent Error, mean ± SD	Percent Absolute Error, Median (IQR)	Exams with Absolute Error less than 20%, n (%)	Exams with Absolute Error 20 to <30%, n (%)	Exams with Absolute Error 30% or more n (%)	Correlation between Z_{efw} and Z_{bw}, r
All Exams							
0-3.9 wks	798	2.9 ± 8.7[a]	5.9 (3.1-9.8)	772 (96.7%)	21 (2.6%)	5 (0.6%)	0.82
4-7.9 wks	705	3.8 ± 10.0[a]	6.5 (3.0-11.3)	658 (93.3%)	37 (5.2%)	10 (1.4%)	0.71
8-11.9 wks	434	4.8 ± 11.4[ab]	7.0 (3.3-11.9)	389 (89.6%)	31 (7.2%)	13 (3.0%)	0.66
12-15.9 wks	262	5.3 ± 12.4[ab]	8.7 (3.7-14.8)[d]	224 (85.5%)	32 (12.2%)	6 (2.3%)	0.55
16-19.9 wks	534	6.4 ± 13.0[abc]	9.4 (4.3-15.4)[d]	457 (86.6%)	55 (10.3%)	22 (4.1%)	0.47
≥ 20 wks	339	5.7 ± 12.0[ab]	8.4 (4.6-13.3)[d]	295 (87.0%)	30 (8.9%)	14 (4.1%)	0.31
Total	3,071	4.5 ± 11.0[a]	7.1 (3.3-12.4)	2,797 (91.0%)	206 (6.7%)	70 (2.3%)	0.65
Last Exam Before Birth							
0-3.9 wks	691	2.8 ± 8.5[a]	5.9 (3.1-9.8)	673 (97.4%)	16 (2.3%)	2 (0.3%)	0.81
4-7.9 wks	155	2.5 ± 8.7[a]	5.7 (2.7-10.2)	151 (97.4%)	4 (2.6%)	0	0.62
8-11.9 wks	44	2.7 ± 9.8[b]	5.9 (2.8-9.4)	41 (93.2%)	3 (6.8%)	0	0.57
12-15.9 wks	23	2.0 ± 12.5[b]	9.1 (3.5-14.6)	20 (87.0%)	3 (13.0%)	0	0.42
16-19.9 wks	136	5.3 ± 12.2[abc]	8.9 (3.1-15.6)[d]	117 (86.0%)	15 (11.0%)	4 (2.9%)	0.36
≥ 20 wks	88	8.7 ± 13.8[abe]	9.3 (5.7-17.2)[d]	67 (76.1%)	14 (15.9%)	7 (8.0%	0.12
Total	1,137	3.5 ± 9.8[a]	6.4 (3.1-11.2)	2,797 (91.0%)	206 (6.7%)	70 (2.3%)	0.71

Latency is the interval between ultrasound and birth.
Correlation column is Pearson correlation coefficient (r) between Z-score of estimated fetal weight and Z-score of birthweight.
a Significantly different than 0, P<0.01, t-test
b Significantly different than 0-3.9 wks group, P<0.01, ANOVA with Sidak test or U-test
c Significantly different than 4-7.9 wks group, P<0.01, ANOVA with Sidak test
d Significantly different than 8-11.9 wks group, U-test
e Significantly different than all groups <16 wks, P<0.05 ANOVA with Sidak test

S1.3 Testing the assumption that z-score of BW equals z-score of EFW

The assumption that z-score of BW should equal the z-score of EFW is assessed in the scatter plot for exams with latencies <12 wks, Figure S1. There is a strong correlation between Z_{efw} and Z_{bw} (r-values 0.82, 0.71, and 0.66 at latencies of 0-3.9 wks, 4-7.9 wks, 8=11.9 wks, respectively). As shown in Table S1, the value of r decreases progressively with increasing latency.

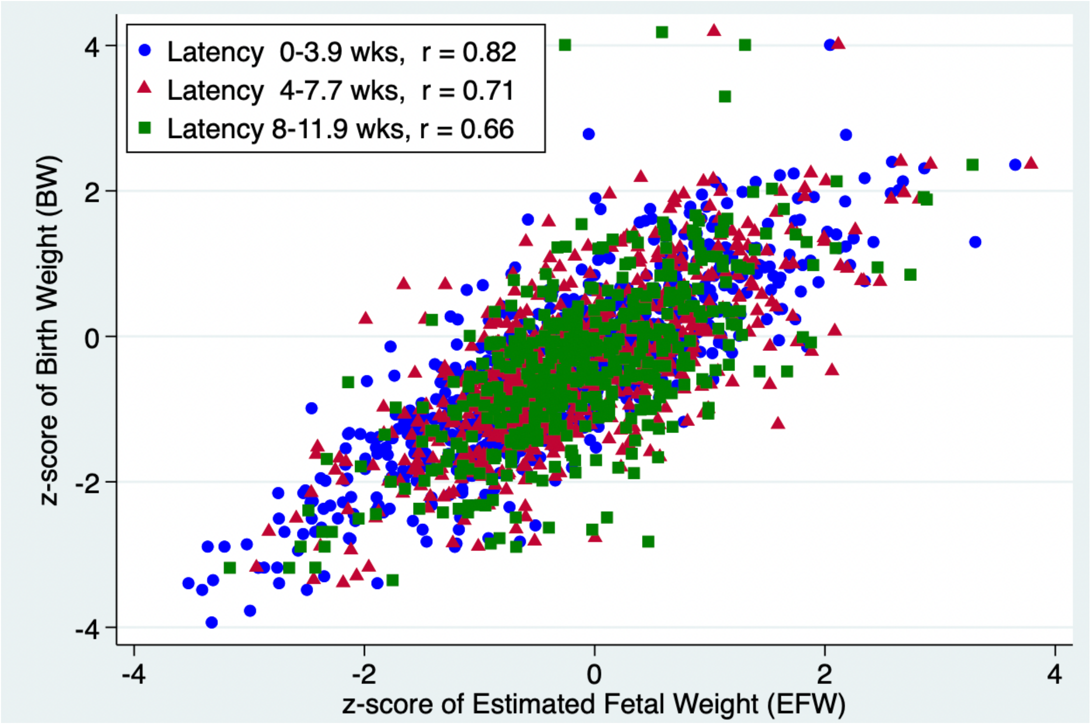

Figure S1. Correlation between z-score of EFW and z-score of BW.
Values of Pearson correlation coefficient (r) are listed at different latencies.

S1.4 References Cited
(numbers refer to main text)

24. Mongelli M, Gardosi J. Gestation-adjusted projection of estimated fetal weight. Acta Obstet Gynecol Scand 1996; 75:28-31.

36. Hadlock FP, Harrist RB, Martinez-Poyer J. In utero analysis of fetal growth: a sonographic weight standard. Radiol 1991; 181:129-133.
