# Supplementary material for: Quantitative Approach to Quality Review of Prenatal Ultrasound Examinations: Estimated Fetal Weight and Fetal Sex
Source: J Clin Med. 2024 Nov 16;13(22):6895. doi: 10.3390/jcm13226895 (PMC11594779; doi:10.3390/jcm13226895)
Supplement: Supplementary file 1 [file jcm-13-06895-s001.zip › S2 - Factors affecting accuracy of BW prediction.pdf]

Supplementary File 2
Exploration of factors potentially affecting accuracy of birth weight prediction

The following analyses are generally restricted to the last ultrasound before birth because overall accuracy was higher for the last exam than for all exams combined.   The exception is when one of the strata has N<100. In such cases all ultrasound exams are analyzed to minimize spurious errors with small N.

Page 2 – Maternal race
Page 3 – Maternal obesity
Page 4 – Maternal age
Page 5 – Newborn sex
Page 6 – Gestational age at time of ultrasound exam
Page 7 – Gestational age at birth
Page 8 – Birth weight
Page 9 – Estimated fetal weight percentile

S2.1 Maternal Race

Table S2.1 shows the accuracy of BW predictions stratified by maternal self-reported race as recorded in the hospital records. We analyzed all 1,938 exams rather than restricting to the last exam before birth because of the small number of Black patients.

Predicted BW was higher than actual BW for all races (positive mean error) but mean error was significantly lower for Hispanic patients than Asian or White patients. This difference did not persist when examining median absolute error, where the Kruskall-Wallace test showed no significant between-group difference (P = 0.13). Black patients had a higher percentage of exams with errors greater than 30% compared to all other groups combined (8.3% vs 1.5%, odds ratio 6.1, 95% CI 1.4-27.3, P <0.05) based on an N of 2.

Table S2.1. Accuracy of birth weight predictions stratified by maternal race, all exams.

Race	N	Percent Error, mean ± SD	Percent Absolute Error, Median (IQR)	Exams with Absolute Error less than 10%, n (%)	Exams with Absolute Error 10 to <20%, n (%)	Exams with Absolute Error 20 to <30%, n (%)	Exams with Absolute Error 30% or more, n (%)
Asian	589	4.4 ± 8.9 [a]	6.1 (2.9-10.8)	423 (71.8%)	135 (22.9%)	29 (4.9%)	2 (0.3%)
Black	24	7.2 ± 14.1 [a]	6.4 (2.3-14.5)	17 (70.8%)	2 (8.3%)	3 (12.5%)	2 (8.3%)
Hispanic	258	2.0 ±10.5 [ab]	6.4 (3.4-11.5)	179 (69.4%)	59 (22.9%)	15 (5.8%)	5 (1.9%)
White	376	4.4 ± 9.0 [a]	6.4 (3.2-10.4)	277 (73.7%)	80 (21.3%)	14 (3.7%)	5 (1.3%)
Other	466	3.5 ± 12.6 [a]	7.6 (3.5-12.8)	143 (63.6%)	59 (26.2%)	15 (6.7%)	8 (3.6%)
Declined	225	3.1 ± 9.2 [a]	6.3 (3.0-10,6)	347 (74.5%)	100 (21.5%)	13 (2.8%)	6 (1.3%)
Total	1,938	3.7 ± 9.9 [a]	6.4 (3.1-11.0) [c]	1386 (71.5%)	435 (22.5%)	89 (4.6%)	28 (1.4%)

"Declined" group includes patients where the race field was recorded as "Declined to State" or was left blank.

a- Significantly different than 0, P < 0.01, t-test

b- Significantly different than Asian or White, P < 0.05, ANOVA with Sidak test

c- No significant between-group difference, P=0.13, Kruskall-Wallace test.

S2.2 Maternal obesity

As shown in Table S2.2, maternal obesity had no significant association with accuracy. We analyzed all 1,938 exams rather than restricting to the last exam before birth because of the small number of obese patients. Obesity was defined based on the indications for the exam as selected by the sonographer, not measurement of height and weight. Because only 6.5% of exams were recorded as Obese, compared to 27.7% of the California population in 2023*, we suspect that obesity was underdiagnosed.

Table S2.2. Accuracy of birth weight predictions in obese and non-obese patients, all exams

Obesity	N	Percent Error, mean ± SD	Percent Absolute Error, Median (IQR)	Exams with Absolute Error less than 10%, n (%)	Exams with Absolute Error 10 to <20%, n (%)	Exams with Absolute Error 20 to <30%, n (%)	Exams with Absolute Error 30% or more, n (%)
Non-obese	1812	3.8 ± 9.9 [a]	6.4 (3.0-11.1)	1,292 (71.3%)	408 (22.55)	85 (4.7%)	27 (1.5%)
Obese	126	2.4 ± 9.5 [a]	6.4 (3.9-10.0)	94 (74.6%)	27 (21.4%)	4 (3.2%)	1 (0.8%)
Total	1,938	3.7 ± 9.9 [a]	6.4 (3.1-11.0) [c]	1,386 (71.5%)	435 (22.5%)	89 (4.6%)	28 (1.4%)

a- Significantly different than 0, P < 0.01, t-test
b- No significant between-group difference, P=0.56, ANOVA
c- No significant between-group difference, P=0.88, Kruskall-Wallace test.

* Centers for Disease Control and Prevention. Adult obesity prevalence maps, 2023. Available at: https://www.cdc.gov/obesity/php/data-research/adult-obesity-prevalence-maps.html, accessed 24 September 2024.

S2.3 Maternal Age

For this analysis, maternal age strata were defined to divide the sample roughly into quartiles. As shown in Table S2.3, predicted BW was higher than BW (positive mean error) in all age strata except ages 15-29 years. There was no significant between-group difference in absolute error (P = 0.25).

Table S2.3. Accuracy of birth weight predictions stratified by maternal age, last exam before birth

Maternal Age, years	N	Percent Error, mean ± SD	Percent Absolute Error, Median (IQR)	Exams with Absolute Error less than 10%, n (%)	Exams with Absolute Error 10 to <20%, n (%)	Exams with Absolute Error 20 to <30%, n (%)	Exams with Absolute Error 30% or more, n (%)
15-29.99	202	0.7 ± 9.3	6.2 (3.1-10.8)	149 (73.7%)	46 (22.8%)	7 (3.5%)	0
30-34.99	321	3.2 ± 8.1 [ab]	5.7 (2.8-9.6)	246 (76.6%)	61 (21.5%)	5 (1.6%)	1 (0.3%)
35-37.99	194	3.1 ± 8.7 [ab]	7.1 (3.6-9.9)	148 (76.3%)	41 (21.3%)	5 (2.6%)	0
≥ 38	173	4.0 ± 8.1 [ab]	5.7 (2.6-10.0)	131 (75.7%)	35 (20.2%)	6 (3.5%)	1 (0.6%)
Total	890	2.8 ± 8.6 [a]	5.8 (3.0-9.9) [d]	674 (75.7%)	191 (21.5%)	23 (2.6%)	2 (0.2%)

a- Significantly different than 0, P < 0.001, t-test
b- Significantly different than 15-29 yr group, P < 0.05, ANOVA with Sidak test
c- No significant between-group difference, P=0.25, Kruskall-Wallace test.

S2.4 Newborn Sex

As shown in Table S2.4, predicted BW was higher than BW (positive mean error) for both females and males, but significantly less so for males. There was no significant between-group difference in absolute error (P = 0.53).

Table S2.4. Accuracy of birth weight predictions in males vs females, last exam before birth

Newborn Sex	N	Percent Error, mean ± SD	Percent Absolute Error, Median (IQR)	Exams with Absolute Error less than 10%, n (%)	Exams with Absolute Error 10 to <20%, n (%)	Exams with Absolute Error 20 to <30%, n (%)	Exams with Absolute Error 30% or more, n (%)
Female	493	3.6 ± 8.7 [a]	5.8 (3.0-10.5)	323 (73.6%)	102 (23.2%)	12 (2.7%)	2 (0.5%)
Male	451	1.9 ± 8.4 [ab]	6.0 (3.0-9.4)	351 (77.8%)	89 (19.7%)	11 (2.4%)	0
Total	890	2.8 ± 8.6 [a]	5.8 (3.0-9.9) [d]	674 (75.7%)	191 (21.5%)	23 (2.6%)	2 (0.2%)

a- Significantly different than 0, P < 0.001, t-test
b- Significantly different than Females, P<0.005, ANOVA
c- No significant between-group difference, P=0.53, Kruskall-Wallace test.

S2.5 Gestational Age at time of Ultrasound Exam

For this analysis, gestational age strata were defined to divide the sample roughly into quartiles. As shown in Table S2.5, when considering all ultrasound exams, those performed before 30 weeks of gestation had larger mean error and median absolute errors than later exams. However, these differences did not persist when the analysis included only the last exam before birth.

Table S2.5. Accuracy of birth weight predictions stratified by gestational age at ultrasound exam, last exam before birth.

Gestational Age, weeks	N	Percent Error, mean ± SD	Percent Absolute Error, Median (IQR)	Exams with Absolute Error less than 10%, n (%)	Exams with Absolute Error 10 to <20%, n (%)	Exams with Absolute Error 20 to <30%, n (%)	Exams with Absolute Error 30% or more, n (%)
All Exams							
18-29.9	415	5.4 ± 12.6 [ab]	7.7 (3.5-13.2) [c]	256 (61.7%)	101 (24.3%)	41 (9.9%)	17 (4.1%)
30-32.9	601	3.4 ± 9.3 [a]	6.2 (3.1-10.9)	434 (72.2%)	137 (22.8%)	26 (4.3%)	4 (0.7%)
33-35.9	433	3.2 ± 9.2 [a]	5.9 (2.6-10.8)	313 (72.3%)	101 (23.3%)	14 (3.2%)	5 (1.2%)
≥ 36	489	3.0 ± 8.2 [a]	6.0 (3.0-9.6)	383 (78.3%)	96 (19.6%)	8 (1.6%)	2 (0.4%)
Total	1,938	3.7 ± 9.9 [a]	6.4 (3.1-11.0)	1,386 (71.5%)	435 (22.5%)	89 (4.6%)	28 (1.4%)
Last Exam Before Birth							
18-29.9	48	1.8 ± 9.8	6.4 (3.0-10.3)	34 (70.8%)	11 (22.9%)	3 (6.3%)	0
30-32.9	140	2.0 ± 8.5 [a]	5.5 (2.9-9.2)	107 (76.4%)	30 (21.4%)	3 (2.1%	0
33-35.9	237	2.9 ± 9.1 [a]	5. 9 (2.9-11.3)	170 (71.7%)	58 (24.5%)	9 (3.8%)	0
≥ 36	465	3.0 ± 8.2 [a]	6.0 (3.0-9.5)	363 (78.1%)	92 (19.85)	8 (1.7%)	2 (0.4%)
Total	890	2.8 ± 8.6 [ad]	5.8 (3.0-9.9) [e]	674 (75.7%)	191 (21.5%)	23 (2.6%)	2 (0.2%)

a- Significantly different than 0, P < 0.01, t-test
b- Significantly different than all other groups, P < 0.05, ANOVA with Sidak test
c- Significantly different than all other groups, P < 0.001, Kruskal-Wallis and U-test
d- No significant between-group difference, P=0.50, ANOVA
e- No significant between-group difference, P=0.67, Kruskal-Wallis test

S2.6 Gestational Age at Birth

For this analysis, gestational age strata were defined to divide the sample roughly into quintiles. As shown in Table S2.6, predicted BW was higher than BW (positive mean error) in all gestational age strata except ≤36 completed weeks. There was no significant between-group difference in mean error or median absolute error.

Table S2.6. Accuracy of birth weight predictions stratified by gestational age at birth, last exam before birth

Gestational Age, completed weeks	N	Percent Error, mean ± SD	Percent Absolute Error, Median (IQR)	Exams with Absolute Error less than 10%, n (%)	Exams with Absolute Error 10 to <20%, n (%)	Exams with Absolute Error 20 to <30%, n (%)	Exams with Absolute Error 30% or more, n (%)
≤36	173	1.1 ± 9.3	5.7 (2.9-10.6)	124 (71.7%)	42 (24.3%)	7 (4.1%)	0
37	144	3.5 ± 8.6 [a]	6.6 (3.0-10.0)	108 (75.0%)	30 (20.8%)	6 (4.2%)	0
38	172	3.2 ± 8.2 [a]	6.7 (3.4-9.5)	131 (76.2%)	38 (22.1%)	3 (1.7%)	0
39	293	3.1 ± 8.4 [a]	5.8 (2.9-9.5)	228 (77.8%)	59 (20.1%)	4 (1.4%)	2 (0.7%)
≥40	108	2.8 ± 8.4 [a]	5.2 (2.7-5.0)	83 (76.9%)	22 (20.4%)	3 (2.8%)	0
Total	890	2.8 ± 8.6 [ab]	5.8 (3.0-9.9) [c]	674 (75.7%)	191 (21.5%)	23 (2.6%)	2 (0.2%)

a- Significantly different than 0, P < 0.01, t-test
b- No significant between-group difference, P=0.07, ANOVA
c- No significant between-group difference, P=0.62, Kruskall-Wallace test

S2.7 Birth Weight

For this analysis, birth weight strata were defined to divide the sample roughly into quartiles. As shown in Table S2.7, mean predicted BW was significantly higher than BW (positive mean error) in all strata except BW ≥3500 gm where mean predicted BW was significantly lower than BW. Median absolute error was significantly higher among those with BW <3200 gm compared to those with BW ≥3200 gm.

Table S2.7. Accuracy of birth weight predictions stratified by birth weight, last exam before birth

Birth Weight, grams	N	Percent Error, mean ± SD	Percent Absolute Error, Median (IQR)	Exams with Absolute Error less than 10%, n (%)	Exams with Absolute Error 10 to <20%, n (%)	Exams with Absolute Error 20 to <30%, n (%)	Exams with Absolute Error 30% or more, n (%)
<2800	241	4.5 ± 9.8 [abc]	7.0 (3.4-12.2) [d]	159 (66.0%)	68 (28.2%)	12 (5.0%)	2 (0.8%)
2800-3199	241	5.0 ± 7.8 [abc]	6.8 (3.3-10.9) [d]	178 (73.9%)	57 (23.7%)	6 (2.5%)	0
3200-3499	199	2.4 ± 6.9 [ab]	4.8 (2.3-8.2)	170 (85.3%)	26 (13.1%)	3 (1.5%)	0
≥3500	209	−1.5 ± 7.8 [ac]	5.1 (2.6-9.2)	167 (79.9%)	40 (19.1%)	2 (1.0%)	0
Total	890	2.8 ± 8.6 [ab]	5.8 (3.0-9.9)	674 (75.7%)	191 (21.5%)	23 (2.6%)	2 (0.2%)

a- Significantly different than 0, P < 0.01, t-test
b- Significantly different than ≥3500 gm group, P<0.001, ANOVA with Sidak test
c- Significantly different than 3200-3400 gm group, P<0.05, ANOVA with Sidak test
d- Significantly different than both groups ≥3200 gm, P<0.001, Kruskal-Wallace and U tests

S2.8 Fetal Weight Percentile

For this analysis, strata were defined by typical clinical groupings, i.e small fetus defined by EFW <10th percentile and large fetus defined by EFW >90th percentile. As shown in Table S2.8, mean error and median absolute error increased progressively as EFW percentile increased when analyzing all exams. When the analysis was restricted to the last exam before birth, the increase in mean error remained significant but the increase in median absolute error did not.

Table S2.8. Accuracy of birth weight predictions stratified by fetal weight percentile, last exam before birth

Fetal Weight Percentile	N	Percent Error, mean ± SD	Percent Absolute Error, Median (IQR)	Exams with Absolute Error less than 10%, n (%)	Exams with Absolute Error 10 to <20%, n (%)	Exams with Absolute Error 20 to <30%, n (%)	Exams with Absolute Error 30% or more, n (%)
All Exams							
< 10th	213	0.3 ± 9.7	6.1 (3.3-9.8)	162 (76.1%)	38 (17.8%)	11 (5.2%)	2 (0.9%)
10th-49th	942	2.9 ± 9.8 [ab]	6.3 (2.9-10.6)	691 (73.4%)	203 (21.6%)	33 (3.5%)	15 (1.6%)
50th-90th	661	5.0 ± 9.6 [abc]	6.5 (3.0-11.3)	452 (68.4%)	168 (25.4%)	32 (4.8%)	9 (1.4%)
>90th	122	7.9 ± 9.5 [abc]	7.7 (3.6-13.6) [d]	81 (66.4%)	26 (21.3%)	13 (10.7%)	2 (1.6%)
Total	1,938	3.7 ± 9.9 [a]	6.4 (3.1-11.0)	1,386 (71.5%)	435 (22.5%)	89 (4.6%)	28 (1.4%)
Last Exam Before Birth							
< 10th	104	–0.6 ± 8.3	5.4 (3.2-8.1)	94 (80.8%)	16 (15.4%)	4 (3.9%)	0
10th-49th	444	2.5 ± 8.8 [ab]	5.9 (2.9-9.6)	339 (76.4%)	94 (21.2%)	9 (2.0%)	2 (0.5%)
50th-90th	293	3.8 ± 8.3 [ab]	6.0 (2.9-10.6)	214 (73.0%)	80 (23.9%)	9 (3.1%)	0
>90th	49	6.5 ± 6.9 [abc]	6.0 (4.7-9.8)	37 (75.7%)	11 (22.5%)	1 (2.0%)	0
Total	890	2.8 ± 8.6 [ab]	5.8 (3.0-9.9) [e]	674 (75.7%)	191 (21.5%)	23 (2.6%)	2 (0.2%)

[a] Significantly different than 0, P < 0.01, t-test
[b] Significantly different than <10th percentile group, P<0.01, ANOVA with Sidak test
[c] Significantly different than 10th to 49th percentile group, P<0.05, ANOVA with Sidak test
[d] Significantly different than both groups <50th percentile, P<0.01, Kruskal-Wallace with U test
[e] No significant difference between groups, P=0.55, Kruskal-Wallace test
