# Supplementary material for: Quantitative Approach to Quality Review of Prenatal Ultrasound Examinations: Estimated Fetal Weight and Fetal Sex
Source: J Clin Med. 2024 Nov 16;13(22):6895. doi: 10.3390/jcm13226895 (PMC11594779; doi:10.3390/jcm13226895)
Supplement: Supplementary file 1 [file jcm-13-06895-s001.zip › S3 - Diagnostic accuracy.pdf]

Supplementary File 3 – Diagnostic Accuracy

S3.1 Fetal growth restriction (FGR) as a predictor of small-for-gestational age (SGA)

We defined FGR using the Society for Maternal-Fetal Medicine (SMFM) definition, that is, either estimated fetal weight (EFW) or fetal abdominal circumference (AC) <10th percentile [3]. For EFW, the 10th percentile was taken as 1.282 standard deviations (SD) below the mean, calculated from formulas Hadlock et al [36]. The 10th percentile for AC was obtained from World Health Organization fetal growth charts, Kiserud et al [18], Table 8, interpolated for exact gestational age. Small-for-gestational age was defined as birth weight (BW) <10th percentile based on USA reference, Duryea et al [37], Table 3.

Test performance characteristics of using FGR to predict SGA are summarized in Table S3.1. Sensitivity was low (51%) when considering the last exam before birth in all 890 patients, but substantially higher (84%) when considering only exams performed <7 days before birth. Specificity was 92% and 83%, respectively. The high area under the receiver operating characteristic curve (ROC) indicates a strong association between EFW z-score and SGA.

Table S3.1 Performance of sonographic fetal growth restriction as a predictor of small-for-gestational age.

	Last Exam Before Birth (N = 890)	Exams ≤7 days Before Birth (N = 175)
Incidence of FGR, n (%)	122/890 (13.7%)	47/175 (26.9%)
Incidence of SGA, n (%)	114/890 (12.8%)	25/175 (14.3%)
True positives, n (%)	58/122 (48%)	21/47 (45%)
False positives, n (%)	64/122 (52%)	26/47 (55%)
True negatives, n (%)	712/768 (92.7%)	124/128 (97%)
False negatives, n (%)	56/768 (7.3%)	4/128 (3%)
Sensitivity, % (95% CI)	51% (48-54%)	84% (79-89%)
Specificity, % (95% CI)	92% (90-94%)	83% (77-88%)
Positive predictive value, % (95% CI)	48% (44-51%)	45% (37-52%)
Negative predictive values, % (95% CI)	93% (91-94%)	78% (94-99%)
Positive likelihood ratio	16.7	4.8
Negative likelihood ratio	0.54	0.19
Odds ratio (95%CI)	11.5 (7.4-18.0)	25.0 (7.9-79.0)
Area under ROC curve	0.88	0.93

SGA - Small for gestational age defined as birth weight <10th percentile using sex-specific United States reference tables [37].
FGR - Fetal growth restriction defined as estimated fetal weight (EFW) or fetal abdominal circumference (AC) <10th percentile [SMFM]
EFW percentiles calculated from formulas in Hadlock et al [36].
AC 10th percentile from Table 8 of World Health Organization Fetal Growth Charts [18]
ROC is receiver operating characteristic curve relating EFW percentile to SGA.

3.2 EFW >90th percentile as a predictor of large-for-gestational age (LGA)

EFW >90th percentile was defined as >1.282 SD above mean, calculated from formulas in Hadlock et al [1991]. LGA was defined as birth weight (BW) >90th percentile based on USA reference, Duryea et al [37], Table 3.

Test performance characteristics of using EFW>90th percentile to predict LGA are summarized in Table S3.2. Sensitivity was low (<50%) when considering either the last exam before birth or exam within 7 days before birth. Specificity was ≥95% in either case. The high area under the receiver operating characteristic curve (ROC) indicates a strong association between EFW z-score and LGA.

Table S3.2 Performance of EFW >90th percentile as a predictor of large-for-gestational age.

	Last Exam Before Birth (N = 890)	Exams ≤7 days Before Birth (N = 175)
Incidence of EFW >90th percentile, n (%)	49/890 (5.5%)	12/175 (6/9%)
Incidence of LGA, n (%)	50/890 (5.6%)	9/175 (5.1%)
True positives, n (%)	21/49 (43%)	4/12 (33%)
False positives, n (%)	28/49 (57%)	8/12 (67%)
True negatives, n (%)	812/841 (96.6%)	158/163 (96.3%)
False negatives, n (%)	29/841 (3.4%)	5/163 (3.7%)
Sensitivity, % (95% CI)	42% (39-45%)	44% (37-52%)
Specificity, % (95% CI)	97% (95-98%)	95% (92-98%)
Positive predictive value, % (95% CI)	43% (40-46%)	33% (26-40%)
Negative predictive values, % (95% CI)	97% (95-98%)	97% (94-99%)
Positive likelihood ratio	12.6	9.2
Negative likelihood ratio	0.60	0.58
Odds Ratio (95% CI)	21.0 (10.7-41.3)	9.9 (2.4-39.8)
Area under ROC curve	0.91	0.95

LGA - Large for gestational age defined as birth weight >10th percentile using sex-specific United States reference tables [37].
Estimated fetal weight (EFW) percentiles calculated from formulas in Hadlock et al [36].
ROC is receiver operating characteristic curve relating EFW percentile to LGA.

References Cited
Numbers refer to main text.

3. Society for Maternal-Fetal Medicine, Martins JG, Biggio JR, Abuhamad A. Society for Maternal-Fetal Medicine Consult Series #52: diagnosis and management of fetal growth restriction. Am J Obstet Gynecol 2020; 223(4): B2-B17.

18. Kiserud T, Piaggio G, Carroli G, Widmer M, Carvalho J, Jensen LN, Giordano D, Cecatti JG, Aleem HA, Talegawkar SA, et al. The World Health Organization Fetal Growth Charts: a multination study of ultrasound biometric measurements and estimated fetal weight. PLoS Med 2017; 14:e1002220.

36. Hadlock FP, Harrist RB, Martinez-Poyer J. In utero analysis of fetal growth: a sonographic weight standard. Radiol 1991; 181:129-133.

37. Duryea EL, Hawkins JS, McIntire DD, Casey BM, Leveno KJ. A revised birth weight reference for the United States. Obstet Gynecol 2014; 124:16-22.
