# Supplementary material for: Quantitative Approach to Quality Review of Prenatal Ultrasound Examinations: Estimated Fetal Weight and Fetal Sex
Source: J Clin Med. 2024 Nov 16;13(22):6895. doi: 10.3390/jcm13226895 (PMC11594779; doi:10.3390/jcm13226895)
Supplement: Supplementary file 1 [file jcm-13-06895-s001.zip › S5 - Sample Stata script, Word version.pdf]

```
* You can copy-all and paste this entire file into Stata’s do-file editor.
* Lines that start with an Asterisk (*) are COMMENTS
* Lines that start without an Asterisk are Stata COMMANDS

cd  "/Users/andrewcombs/Desktop/Analysis/EFW Accuracy Sample/"
* cd means Change Directory - this will become the default folder for finding and saving files
* You will NEED TO CUSTOMIZE THIS to match the file structure in your computer.

log using "LOG EFW accuracy.smcl", replace
* the log file is where results are stored

cd  "/Users/andrewcombs/Desktop/Analysis/EFW Accuracy Sample/"
version 13
* the script will work for Stata version 13 and higher.
* it may work for earlier versions as well, but has not been tested
set more off

import excel "S4 - EXCEL Exams w BW pseudodata.xlsx", firstrow clear
format Notes %-18s
list Notes if Notes !="", noobs sep(30)
drop Notes

** This section is for definition and renaming of variables
* shorter variable name means less typing, less chance for typos!
* the variables in the Excel file were named by Viewpoint
rename PersonNumber Person
generate MatAge=(AssignedEDD-DOB)/365.25
drop DOB
* calculates MaternalAge at due date and removes DOB from analysis file (PHI)
generate GAus=40-(AssignedEDD-Examdate)/7
generate GAdel=40-(AssignedEDD-DelivDate)/7
* GAus is Gestational Age on the date of ultrasound exam, GAdel is GA at delivery
rename BPDmm BPD
rename HCmm HC
rename ACmm AC
rename FLmm FL
replace BPD=BPD/10
replace HC= HC/10
replace AC=AC/10
replace FL=FL/10
* Dividing by 10 converts the biometry measures to CM rather than MM
rename Gender uSex
rename nbSex nSex
* uSex is sex determined by ultrasound, nSex is sex assigned at birth
```

```
tabulate uSex nSex
replace uSex=upper(substr(uSex,1,1))
* recodes uSex as first letter only, upper case
tabulate uSex nSex
rename Sonographer Tech
rename ReadingPhysician Doc


**** CASE COUNTS for Figure 1 Flow Chart, twins, stillbirths
tabulate CardiacActivity
drop if trim(CardiacActivity)=="Absent"
* deletes cases with Absent fetal heart beat on ultrasound.
tabulate Live
drop if Live=="N"
* deletes stillbirths
summarize BWtwinB
drop if BWtwinB <.
* deletes twin pregnancies
drop CardiacActivity Live BWtwinB

** this section generates z-scores for biometry.
* For BPD, z is base on Hadlock 1985
* For other biometry z is based on formulas derived from World Health Org Fetal Growth Charts
* In the formulas, m indicates mean normal value at GAus, s indicates SD, z indicates z-score.
generate mBPD= -3.08 + 0.41*GAus - 0.000061*GAus^3
generate sBPD=0.3
generate zBPD = (BPD-mBPD)/sBPD
gen mHC = -96.88203 +13.58783*GAus +0.0654288*GAus^2 -0.0033107*GAus^3
* typo in cubic term as published + sign instead of minus
gen sHC = 4.849937 -0.0297189*GAus +0.012416*GAus^2 -0.0001731*GAus^3
gen zHC = (10*HC-mHC)/sHC
gen mAC = 0.0021842*GAus^3 - 0.2334435*GAus^2 + 18.33569*GAus -145.2554
gen sAC = 0.0005526*GAus^ -0.0300764*GAus^2 +0.9048439*GAus  -11.0689
gen zAC = (10*AC-mAC)/sAC
gen mFL = -44.99124 +4.795792*GAus -0.0498716*GAus^2 +0.0001113*GAus^3
*typo in square term published - missing 0
gen sFL = 1.710339 -0.045024*GAus  +0.0037156*GAus^2 -0.000046*GAus^3
*typo in square term published (extra 1)
gen zFL = (10*FL-mFL)/sFL
* list cases with any z-score more than 5 SD from the mean, for auditing chart
list ExamID Person Examdate zAC zHC zBPD zFL if abs(zBPD)>5 | abs(zHC)>5 | abs(zAC)>5 | abs(zFL)>5, noobs
* drop cases with any z-score more than 6 SD from mean
drop if abs(zBPD)>6 | abs(zHC)>6 | abs(zAC)>6 | abs(zFL)>6
drop mBPD mHC mAC mFL sBPD sHC sAC sFL
```

```
*** Drop Exams w implausible data, typically where US and delivery are different pregnancies (Latency <0 or GA >50)***
generate Latency=(DelivDate-Examdate)/7
list ExamID Examdate DelivDate GAus GAdel if GAdel>50, noobs
list Person Examdate DelivDate Latency if (Latency<0), noobs
drop if Latency<0
drop if GAdel >50

*** Save Date File
save "DATA Exams w BW.dta", replace
* excludes stillbirths, multifetal, extreme outlier case
use "DATA Exams w BW.dta" , clear
save temp.dta, replace
* temp.dta is working copy
* Using working copy prevents accidentally overwriting a clean data file by issuing an unintentional Save command.

generate Latgroup=""
* Latgroup is a categorical grouping of Latency
replace Latgroup="20+ wks    " if Latency<.
replace Latgroup="16-19.9 wks" if Latency<20
replace Latgroup="12-15.9 wks" if Latency<16
replace Latgroup=" 8-11.9 wks" if Latency<12
replace Latgroup=" 4- 7.9 wks" if Latency<8
replace Latgroup=" 0- 3.9 wks" if Latency<4
tabulate Latgroup

** EFW using Hadlock 3-parameter formula
generate EFW = 10^(1.326 -0.00326*AC*FL + 0.0107*HC + 0.0438*AC + 0.158*FL)
* BWpred is predicted BW using formula in article, as derived in Supplementary File 1.
generate BWpred = EFW * exp(-0.00354*(GAdel^2-GAus^2) + 0.332*(GAdel-GAus))
* Error (Err) and Absolute Error (absErr) are expressed as percent of birth weight (BW)
generate Err=100*(BWpred-BW)/BW
generate absErr=abs(Err)

generate Errgroup=""
* Errgroup groups is a categorical grouping of absErr
replace Errgroup = "> 40%"  if absErr<.
replace Errgroup = "30-40%" if absErr<=40
replace Errgroup = "20-30%" if absErr<=30
replace Errgroup = "10-20%" if absErr<=20
replace Errgroup = "0-10%"  if absErr<=10

** Define Last Exam
* LastExam is a logical variable, 1 if last exam for a given patient, otherwise 0.
```

```
generate LastExam=0
bysort Person: egen MaxExam=max(Examdate)
replace LastExam=1 if Examdate==MaxExam

* Compute z-score of BW (zBW) and z-score of EFW (zEFW)
* Mean and SD of standard BW from Hadlock 1991
generate MBW= exp(0.578 + 0.332*GAdel - 0.00354*GAdel^2)
generate SBW= 0.12*MBW
generate zBW= (BW-MBW)/SBW
* Mean and SD of standard EFW from Hadlock 1991
generate MFW= exp(0.578 + 0.332*GAus - 0.00354*GAus^2)
generate SFW= 0.12*MFW
generate zEFW= (EFW-MFW)/SFW

*** RESULTS

**Text Section 3.2 DELIVERIES WITHIN ONE DAY
* Latency <0.2 weeks is same as Latency 0 days or 1 day
generate gmErr=EFW-BW
summarize gmErr if Latency<0.2, detail
oneway gmErr Latency if Latency<0.2, tabulate
summarize Err if Latency<0.2
summarize absErr if Latency<0.2, detail
tab Errgroup if Latency<0.2
ttest gmErr==0 if Latency<0.2
ttest Err==0 if Latency<0.2
summarize BW if Latency<0.2
summarize GAdel if Latency<0.2

**** TABLES
* The same basic scheme is used for Tables 1,2,and 4 in the main article and most of the supplementary tables
* We annotate the commands for the first table, but do not repeat the annotations for subsequent tables.
* We presuent supplementary Table S1.1 first because it is the only one that includes Latency ≥12 weeks.
* After that, exams with Latency ≥12 weeks are dropped.

*** SUPPLEMENTARY TABLE S1.1

** COLUMNS 1 & 2

* "oneway" is the one-way ANOVA with Sidak multiple compoarisons test for the column Percent Error.
oneway Err Latgroup, tabulate sidak
* The first table shows N, mean and SD for each group.
* "Prob > F" is listed as 0.0000 meaning P<0.0001 for the overall between-groups difference.
* The bottom table (Sidak) shows all possible pairwise comparisons.
```

```
* For example, the comparison between the groups 0-3.9 wks vs 12-15.9 wks is signif at P=0.025
*       and the comparison between groups 4-7.9 wks vs 12-15.9 wks is not signif (P=0.457)

* The t-tests below are used for the footnotes as to whether mean Err was different than 0.
bysort Latgroup: ttest Err==0
ttest Err==0
* The bottom line of the t-test result shows 3 p-values.  The one in the middle is the 2-tailed value.
* The left and right p-values are one-tailed tests, asking whether mean is less than 0 or more than 0.
* We don't have an a priori reason to think that mean Error would be above or below 0
* Therefore, the 2-tailed test is appropriate.

** COLUMN 3

* The "summarize" command reports the 25th, 50th, and 75th percentile for the column Percent Absolute Error
*     (plus a ton of other information)
* The first one summarizes the whole data set, and the second one divides the summary by Latency group
summarize absErr, detail
bysort Latgroup: summarize absErr, detail

kwallis absErr, by(Latgroup)
* The non-parametric Kruskal-Wallace tests the signficance of betweeen-grooup differences in AbsErr.
* Here "Prob = 0.0001" means there is some highly significant between-group difference.

* If the KW shows signficance, then pairwise ranksum tests (U-test) were used (see below)
* I did not run ranksums on every possible pair.  I started with pairs with large differences.
* Once I found a non-significant pair, it seemed obvious the pairs with smaller difference
*   would also likely be non-signifcant.
* You will NEED TO CUSTOMIZE: decide which pairs to compare and edit the "if" statements below
* In stata the ampersand (&) is used for "and" and the vertical bar (|) means "or"
ranksum absErr if Latency <8, by(Latgroup)
ranksum absErr if Latency <4 | (Latency >=8 & Latency<12), by(Latgroup)
ranksum absErr if (Latency >=12 & Latency<16) | (Latency >=8 & Latency<12), by(Latgroup)
ranksum absErr if (Latency >=16 & Latency<20) | Latency >=20 , by(Latgroup)

* COLUMNS 4-6
* This tabulation shows the N and percent of exams with various amounts of absolute error

tabulate Latgroup Errgroup, row nokey

* COLUMN 7
* The "regress" command was used to find r-squared values.
* r was calculated from r-squared manually, r = square root of r-squared.

regress zBW zEFW
```

```
bysort Latgroup: regress zBW zEFW

* REPEAT FOR LAST EXAM ONLY (lower half of table)
oneway Err Latgroup if LastExam, tabulate sidak
bysort Latgroup: ttest Err==0 if LastExam
ttest Err==0 if LastExam
bysort Latgroup: summarize absErr if LastExam, detail
summarize absErr if LastExam, detail
kwallis absErr if LastExam, by(Latgroup)
ranksum absErr if LastExam & (Latgroup=="12-15.9 wks" | Latgroup==" 8-11.9 wks"), by(Latgroup)
ranksum absErr if LastExam & (Latgroup=="16-19.9 wks" | Latgroup==" 8-11.9 wks"), by(Latgroup)
ranksum absErr if LastExam & (Latgroup=="20+ wks    " | Latgroup==" 8-11.9 wks"), by(Latgroup)
tabulate Latgroup Errgroup if LastExam, row nokey
regress zBW zEFW if LastExam
bysort Latgroup: regress zBW zEFW if LastExam

****** REMAINDER OF THE ANALYSIS WILL ONLY INCLUDE EXAMS WITH LATENCY <12 wks.
drop if Latency >=12

save "DATA Analysis Set.dta", replace
use "DATA Analysis Set.dta", clear
save temp.dta, replace


*** TABLE 1 ACCURACY STRATIFIED BY LATENCY GROUP, upper half of table
oneway Err Latgroup, tabulate sidak
bysort Latgroup: ttest Err==0
summarize Err
ttest Err==0
bysort Latgroup: summarize absErr, detail
summarize absErr, detail
kwallis absErr, by(Latgroup)
ranksum absErr if Latency <8, by(Latgroup)
ranksum absErr if Latency >=4, by(Latgroup)
ranksum absErr if Latency <4 | Latency >=8, by(Latgroup)
tabulate Latgroup Errgroup, row nokey

* Lower half of Table 1
oneway Err Latgroup if LastExam, tabulate sidak
bysort Latgroup: ttest Err==0 if LastExam
summarize Err  if LastExam
ttest Err==0  if LastExam
bysort Latgroup: summarize absErr  if LastExam, detail
summarize absErr if LastExam, detail
```

```
kwallis absErr if LastExam, by(Latgroup)
ranksum absErr if Latency <8 & LastExam, by(Latgroup)
ranksum absErr if Latency >=4 & LastExam, by(Latgroup)
ranksum absErr  if LastExam & (Latency <4 | Latency >=8), by(Latgroup)
tabulate Latgroup Errgroup  if LastExam, row nokey


** TABLE 2 RESULTS STRATIFIED BY SONOGRAPHER, upper half of table
bysort Tech: generate NtExams=_N
* NtExams is the number of exams performed for each sonographer
generate Threshold=100
* Threshold is the minimum number of exams performed by a sonographer to be included in Table 2.
* We used 100, but YOU CAN CUSTOMIZE TO ANY OTHER NUMBER

oneway Err Tech if NtExams>Threshold, tabulate sidak
* The Total row in the ANOVA table above shows mean and SD for only the Techs listed.
* The summarize cammand below shows the mean and SD for all techs.

summarize Err
ttest Err==0
bysort Tech: ttest Err==0 if NtExams>Threshold
summarize absErr, detail
bysort Tech: summarize absErr if NtExam>Threshold, detail
kwallis absErr if NtExam>Threshold, by(Tech)
* The p-value in the pseudodata is shown as "Prob = 0.0987" meaning no signif difference in AbsErr between techs.
* If it had been significant, you would need to do 2-way comparisons to find signif between-tech differences.
* An example two-way comparison is next, using Rank-Sum to do Mann-Whitney U test (also known as Wilcoxon)
* Rather than type in the full name of the Tech, it uses the substring function for the first 3 characters of the name.

ranksum absErr if substr(Tech,1,3)=="Nat" | substr(Tech,1,3)=="Ver", by(Tech)
* The 2-way comparison is also not significant:  "Prob >|z| = 0.1350"
* The Note indicates that this is an approximate p-values
* The Note suggests running the test aaain using the "exact" option

ranksum absErr if substr(Tech,1,3)=="Nat" | substr(Tech,1,3)=="Ver", by(Tech) exact
* Not much difference!

tabulate Tech Errgroup if NtExams>Threshold, row nokey
* The Total row in the table above includes only the listed Sonographers.
* The tabulation below gives the totals for thw whole practice.

tab Errgroup

* Lower half of Table
```

```
oneway Err Tech if NtExams>Threshold & LastExam, tabulate sidak
summarize Err if LastExam
ttest Err==0 if LastExam
bysort Tech: ttest Err==0 if NtExams>Threshold & LastExam
bysort Tech: summarize absErr if NtExams>Threshold & LastExam, detail
summarize absErr if LastExam, detail
kwallis absErr if NtExam>Threshold & LastExam, by(Tech)
tab Tech Errgroup if NtExams>Threshold & LastExam, row nokey
tab Errgroup if LastExam

** TABLE 3 LATENCY AND BIOMETRY z-SCORES BY SONOGRAPHER

oneway Latency Tech if NtExams>Threshold, tabulate sidak
summarize Latency
oneway zHC Tech if NtExams>Threshold, tabulate sidak
summarize zHC
ttest zHC==0
egen zHCmean=mean(zHC)
* zHCmean defined above is the mean z-score of HC for the entire practice
* The t-test below tests whether each Tech's mean z-score is different than practice-wide mean

bysort Tech: ttest zHC==zHCmean if NtExams>Threshold

oneway zAC Tech if NtExams>Threshold, tabulate sidak
summarize zAC
ttest zAC==0
egen zACmean=mean(zAC)
bysort Tech: ttest zAC==zACmean if NtExams

oneway zFL Tech if NtExams>Threshold, tabulate sidak
summarize zFL
ttest zFL==0
egen zFLmean=mean(zFL)
bysort Tech: ttest zFL==zFLmean if NtExams>Threshold

*** TABLE 4 Accuracy by Doc
oneway Err Doc, tabulate sidak
summarize Err
kwallis absErr, by(Doc)
bysort Doc: summarize absErr, detail
tabulate Doc Errgroup, row nokey

oneway Err Doc if LastExam, tabulate sidak
summarize Err if LastExam
```

```
kwallis absErr if LastExam, by(Doc)
bysort Doc: summarize absErr if LastExam, detail
tabulate Doc Errgroup if LastExam, row nokey


*** AUDIT CASES with Absolute Error >30%
sort Person Examdate
gen GAu=round(GAus)
gen GAd=round(GAdel)
gen Lat=round(Latency)
gen BWp=round(BWpred)
gen rErr=round(Err)
list Person Examdate GAu DelivDate GAd Lat BW BWp rErr if (absErr>30), noobs
* The table above is a list of exams with absolute Error >30%
* To review images in Viewpoint, we manually search by PersonNumber then find the matching exam date
* GAu is ultrasound GA, GAd is delivery GA, Lat is latency
* BWp is predicted BW, and rErr is error
* All values are rounded to integers to keep the table readable.
* For all other analyses, the exact values are used, not rounded.
* The rounded values are deleted on the next line.
drop GAu GAd Lat BWp rErr


* TABLE 5 - FETAL SEX ACCURACY
sort Person Examdate
list Person ExamID Examdate DelivDate uSex nSex ///
       if  (trim(uSex)=="M" & trim(nSex)=="F") ///
        |  (trim(uSex)=="F" & trim(nSex)=="M"), noobs
* Table shows cases where fetal Sex (ultrasound) disagreed with newborn sex.
* We list only exams where sex was reported on ultrasound.
* We don't reassess sex on every exam, so many exams had no listing for gender

list Person ExamID Examdate DelivDate uSex nSex Tech if trim(uSex)=="U"
* Table shows all exams where Gender was listed as Unknown, meaning that imaging was suboptimal.



*** SUPPLEMENTARY FILE 2

* ACCURACY BY NEWBORN SEX
oneway Err nSex, tabulate sidak
bysort nSex: ttest Err==0
ttest Err, by(nSex)
kwallis absErr, by(nSex)
```

```
ranksum absErr, by(nSex)
bysort nSex: summarize absErr, detail
tabulate nSex Errgroup, row nokey

oneway Err nSex if LastExam, tabulate sidak
bysort nSex: ttest Err==0 if LastExam
kwallis absErr if LastExam, by(nSex)
ranksum absErr if LastExam, by(nSex)
bysort nSex: summarize absErr if LastExam, detail
tabulate nSex Errgroup if LastExam, row nokey

* ACCURACY BY MATERNAL AGE GROUP
generate Agegroup=""
replace Agegroup="38+" if MatAge<.
replace Agegroup="35-37.99" if MatAge<38
replace Agegroup="30-34.99" if MatAge<35
replace Agegroup="15-29.9" if MatAge<30
oneway Err Agegroup, tabulate sidak
bysort Agegroup: ttest Err==0
kwallis absErr, by(Agegroup)
bysort Agegroup: summarize absErr, detail
tabulate Agegroup Errgroup, row nokey

oneway Err Agegroup if LastExam, tabulate sidak
bysort Agegroup: ttest Err==0 if LastExam
kwallis absErr if LastExam, by(Agegroup)
bysort Agegroup: summarize absErr if LastExam, detail
tabulate Agegroup Errgroup if LastExam, row nokey

* ACCURACY BY GA AT ULTRASOUND
generate GUgroup=""
replace GUgroup="36+" if GAus<.
replace GUgroup="33-35.9" if GAus<36
replace GUgroup="30-32.9" if GAus<33
replace GUgroup="18-29.9" if GAus<30

oneway Err GUgroup, tabulate sidak
bysort GUgroup: ttest Err==0
kwallis absErr, by(GUgroup)
ranksum absErr if GUgroup=="18-29.9" | GUgroup=="30-32.9", by(GUgroup)
ranksum absErr if GUgroup=="33-35.9" | GUgroup=="30-32.9", by(GUgroup)
bysort GUgroup: summarize absErr, detail
tabulate GUgroup Errgroup, row nokey
```

```
oneway Err GUgroup if LastExam, tabulate sidak
bysort GUgroup: ttest Err==0 if LastExam
kwallis absErr if LastExam, by(GUgroup)
bysort GUgroup: summarize absErr if LastExam, detail
tabulate GUgroup Errgroup if LastExam, row nokey

* ACCURACY BY GA AT BIRTH
generate tGA=trunc(GAdel)
generate GDgroup=""
replace GDgroup="40+" if tGA<.
replace GDgroup="39 " if tGA==39
replace GDgroup="38 " if tGA==38
replace GDgroup="37 " if tGA==37
replace GDgroup="36-" if GAdel<37

oneway Err GDgroup, tabulate sidak
bysort GDgroup: ttest Err==0
kwallis absErr, by(GDgroup)
bysort GDgroup: summarize absErr, detail
tabulate GDgroup Errgroup, row nokey

oneway Err GDgroup if LastExam, tabulate sidak
bysort GDgroup: ttest Err==0 if LastExam
kwallis absErr if LastExam, by(GDgroup)
bysort GDgroup: summarize absErr if LastExam, detail
tabulate GDgroup Errgroup if LastExam, row nokey

*** ACCURACY BY BIRTH WEIGHT GROUP
generate BWgroup=""
replace BWgroup="3500+    " if BW<.
replace BWgroup="3200-3499" if BW<3500
replace BWgroup="2800-3199" if BW<3200
replace BWgroup="0415-2799" if BW<2800

oneway Err BWgroup, tabulate sidak
bysort BWgroup: ttest Err==0
kwallis absErr, by(BWgroup)
bysort BWgroup: summarize absErr, detail
ranksum absErr if BWgroup=="0415-2799"| BWgroup=="2800-3199", by(BWgroup)
ranksum absErr if BWgroup=="3200-3499"| BWgroup=="2800-3199", by(BWgroup)
ranksum absErr if BWgroup=="3200-3499"| BWgroup=="3500+    ", by(BWgroup)
tabulate BWgroup Errgroup, row nokey

oneway Err BWgroup if LastExam, tabulate sidak
```

```
bysort BWgroup: ttest Err==0 if LastExam
kwallis absErr if LastExam, by(BWgroup)
bysort BWgroup: summarize absErr if LastExam, detail
ranksum absErr if (BWgroup=="0415-2799"| BWgroup=="2800-3199") & LastExam, by(BWgroup)
ranksum absErr if (BWgroup=="3200-3499"| BWgroup=="2800-3199") & LastExam, by(BWgroup)
ranksum absErr if (BWgroup=="3200-3499"| BWgroup=="3500+    ") & LastExam, by(BWgroup)
tabulate BWgroup Errgroup if LastExam, row nokey

* ACCURACY BY EFW PERCENTILE GROUPING
generate EFWgroup=""
replace EFWgroup="90+  " if zEFW<.
replace EFWgroup="50-90" if zEFW<1.282
replace EFWgroup="10-49" if zEFW<0
replace EFWgroup="10-  " if zEFW<-1.282

oneway Err EFWgroup, tabulate sidak
bysort EFWgroup: ttest Err==0
kwallis absErr, by(EFWgroup)
bysort EFWgroup: summarize absErr, detail
ranksum absErr if EFWgroup=="90+  "| EFWgroup=="50-90", by(EFWgroup)
ranksum absErr if EFWgroup=="90+  "| EFWgroup=="10-49", by(EFWgroup)
ranksum absErr if EFWgroup=="10-  "| EFWgroup=="50-90", by(EFWgroup)
ranksum absErr if EFWgroup=="10-  "| EFWgroup=="10-49", by(EFWgroup)
tabulate EFWgroup Errgroup, row nokey

oneway Err EFWgroup if LastExam, tabulate sidak
bysort EFWgroup: ttest Err==0 if LastExam
kwallis absErr if LastExam, by(EFWgroup)
bysort EFWgroup: summarize absErr if LastExam, detail
tabulate EFWgroup Errgroup if LastExam, row nokey

* Sample psuedodata file does not contain maternal race or obesity data,
* If you want to include these fields, you cah write analogous blocks for these.


*** SUPPLEMENTARY FILE 3 DIAGNOSTIC ACCURACY FGR, LGA

*** AC 1Oth percentile Kisered WHO PLoSMed 2017;14:e1002220, table 8
gen tGAus=trunc(GAus)
matrix kAC10 = (23,24,25,26,27,28,29,30,31,32,33,34,35,36,37,38,39,40,41,42 \    ///
       173,184,195,505,215,225,234,243,252,260,269,277,286,294,304,313,324,335,344,353)
gen lower10ac = kAC10[2,tGAus-22]
gen upper10ac = kAC10[2,tGAus-21]
gen fxnwkUS=GAus-tGAus
```

```
gen AC10th = lower10ac+fxnwk*(upper10ac-lower10ac)
gen smallACk=0
replace smallACk=1 if 10*AC<AC10th

**** Duryea Birthweight centiles (from Table 3 in Obstet Gynecol 2014; 124:16-22)
* 10th percentile cutoffs
matrix DurF10 = (23,24,25,26,27,28,29,30,31,32,33,34,35,36,37,38,39,40,41,42 \    ///
              530,545,567,622,702,800,911,1033,1173,1335,1536,1747,1987,2230,2461,2664,2829,2950,3020,3033)
gen lower10 = DurF10[2,tGA-22] if nSex=="F"
gen upper10 = DurF10[2,tGA-21] if nSex=="F"
matrix DurM10 = (23,24,25,26,27,28,29,30,31,32,33,34,35,36,37,38,39,40,41,42 \    ///
              570,580,595,652,741,851,972,1102,1247,1414,1608,1834,2078,2325,2560,2766,2935,3062,3143,3175)
replace lower10 = DurM10[2,tGA-22] if nSex=="M"
replace upper10 = DurM10[2,tGA-21] if nSex=="M"
gen fxnwk=GAdel-tGA
gen centile10 = lower10+fxnwk*(upper10-lower10)
generate DurSGA=0
replace DurSGA=1 if BW<centile10
*list GAdel nSex lower10 centile10 upper10 BW DurSGA in 1/15

*90th percentile cutoffs
matrix DurF90 = (23,24,25,26,27,28,29,30,31,32,33,34,35,36,37,38,39,40,41,42 \    ///
              750,820,912,1047,1217,1410,1616,1831,2055,2291,2540,2801,3063,3311,3522,3714,3856,3973,4082,4198)
gen lower90 = DurF90[2,tGA-22] if nSex=="F"
gen upper90 = DurF90[2,tGA-21] if nSex=="F"
matrix DurM90 = (23,24,25,26,27,28,29,30,31,32,33,34,35,36,37,38,39,40,41,42 \    ///
              770,855,964,1110,1284,1479,1686,1901,2128,2367,2622,2892,3165,3426,3661,3856,4010,4135,4242,4345)
replace lower90 = DurM90[2,tGA-22] if nSex=="M"
replace upper90 = DurM90[2,tGA-21] if nSex=="M"
gen centile90 = lower90+fxnwk*(upper90-lower90)
generate DurLGA=0
replace DurLGA=1 if BW>centile90

** Diagnostic Performance
generate FGR=0
replace FGR=1 if zEFW<-1.282
replace FGR=1 if smallACk
generate LGU=0
replace LGU=1 if zEFW>1.282 & zEFW<.

diagtest FGR DurSGA if LastExam
diagtest FGR DurSGA if Latency<1
diagtest LGU DurLGA if LastExam
diagtest LGU DurLGA if Latency<1
```

```
* Areas under ROC curves obtained at end of file
* Likelihood ratios and odds ratios calculated offline manually for tables in artice.


**********  GRAPHS  *********

* You will NEED TO CUSTOMIZE the directory specification in next line
cd  "/Users/andrewcombs/Desktop/Analysis/EFW Accuracy Sample/Graphs

*** FOR Suppl 1 - Validation of Centile projection
bysort Latgroup: regress zBW zEFW
regress zBW zEFW
twoway (scatter zBW zEFW if Latency<4, mcolor(blue) msymbol(o)) ///
              (scatter zBW zEFW if Latency<8 & Latency >=4, mcolor(cranberry) msymbol(t)) ///
              (scatter zBW zEFW if Latency<12 & Latency>=8, mcolor(green) msymbol(s) ///
              legend(ring(0) pos(11) col(1) ///
                     lab(3 "Latency 8-11.9 wks, r = 0.66") ///
                     lab(2 "Latency  4-7.7 wks,  r = 0.71") ///
                     lab(1 "Latency  0-3.9 wks,  r = 0.82"))  ///
              ytitle("z-score of Birth Weight (BW)") ///
              ylabel(,angle(0))  ///
              note("Entire cohort, n = 1736")  ///
              xtitle("z-score of Estimated Fetal Weight (EFW)"))
graph save Graph "GRAPH zEFW vs zBW by Latency.gph"       , replace

*** Last Exam only
bysort Latgroup: regress zBW zEFW if LastExam
regress zBW zEFW if LastExam

twoway (scatter zBW zEFW if Latency<4 & LastExam, mcolor(blue) msymbol(o)) ///
       (scatter zBW zEFW if Latency<8 & Latency >=4 & LastExam, mcolor(cranberry) msymbol(t)) ///
       (scatter zBW zEFW if Latency<12 & Latency>=8 & LastExam, mcolor(green) msymbol(s) ///
              legend(ring(0) pos(11) col(1) ///
                     lab(3 "Latency 8-11.9 wks, r = 0.57") ///
                     lab(2 "Latency  4-7.7 wks,  r = 0.62") ///
                     lab(1 "Latency  0-3.9 wks,  r = 0.81"))  ///
              ytitle("z-score of Birth Weight (BW)") ///
              ylabel(,angle(0))  ///
              note("Last exam before birth, n = 815")  ///
              xtitle("z-score of Estimated Fetal Weight (EFW)"))
graph save Graph "GRAPH zEFW vs zBW by Latency Last Exam.gph"    , replace

**** ROC Plots
```

```
* mzEFW is negative of zEFW so SGA ROCs run in correct direction
generate mzEFW=0-zEFW

logistic DurSGA mzEFW if LastExam
lroc
graph save Graph "GRAPH roc Duryea SGA vs zEFW lastexam.gph", replace

logistic DurSGA mzEFW if Latency<1
lroc
graph save Graph "GRAPH roc Duryea SGA vs zEFW latency<7.gph", replace

logistic DurLGA zEFW if LastExam
lroc
graph save Graph "GRAPH roc Duryea LGA vs zEFW lastexam.gph", replace

logistic DurLGA zEFW if Latency<1
lroc
graph save Graph "GRAPH roc Duryea LGA vs zEFW latency<7.gph", replace


cd  "/Users/andrewcombs/Desktop/Analysis/EFW Accuracy Sample/
save ztemp4.dta, replace
log close
exit
```
